# The comparative efficacy of angiosome-directed and indirect revascularisation strategies to aid healing of chronic foot wounds in patients with co-morbid diabetes mellitus and critical limb ischaemia: a literature review

**DOI:** 10.1186/s13047-017-0206-5

**Published:** 2017-06-28

**Authors:** Benedictine Y. C. Khor, Pamela Price

**Affiliations:** 1 Department of Podiatry, Galloway Community Hospital, NHS Dumfries & Galloway, Stranraer, UK; 2 Department of Podiatry, Queen Elizabeth University Hospital, NHS Greater Glasgow & Clyde, Glasgow, UK

**Keywords:** Angiosome, Critical limb ischaemia, Diabetic foot, Peripheral vascular disease, Revascularisation, Wound healing

## Abstract

**Background:**

Ischaemic ulcerations have been reported to persist and/or deteriorate despite technically successful revascularisations; a higher incidence of which affects patients with diabetes and critical limb ischaemia. In the context of wound healing, it is unclear if applications of the angiosome concept in ‘direct revascularisation’ (DR) would be able to aid the healing of chronic foot ulcerations better than the current ‘best vessel’ or ‘indirect revascularisation’ (IR) strategy in patients with co-morbid diabetes and critical limb ischaemia.

**Methods:**

A literature search was conducted in eight electronic databases, namely AMED, CINAHL, The Cochrane Library, ProQuest Health & Medicine Complete, ProQuest Nursing & Allied Health Source, PubMed, ScienceDirect and TRIP database. Articles were initially screened against a pre-established inclusion and exclusion criteria to determine eligibility and subsequently appraised using the Newcastle-Ottawa Scale.

**Results:**

Five retrospective studies of varying methodological quality were eligible for inclusion in this review. Critical analysis of an aggregated population (*n* = 280) from methodologically stronger studies indicates better wound healing outcomes in subjects who had undergone DR as compared to IR (*p* < 0.001; *p* = 0.04). DR also appears to result in a nearly twofold increase in probability of wound healing within 12 months (hazard ratio, 1.97; 95% CI, 1.34–2.90). This suggests that achieving direct arterial perfusion to the site of ulceration may be important for the healing of chronic diabetic foot ulcerations.

**Conclusion:**

Incorporating an angiosome-directed approach in the lower limb revascularisation strategy could be a very useful adjunct to a solely indirect approach, which could increase the likelihood of wound healing. With the limited data currently available, findings appear promising and merit from further investigation. Additional research to form a solid evidence base for this revised strategy in patients with co-morbid diabetes and critical limb ischaemia is warranted.

**Electronic supplementary material:**

The online version of this article (doi:10.1186/s13047-017-0206-5) contains supplementary material, which is available to authorized users.

## Background

### Critical limb ischaemia

Critical limb ischaemia (CLI) represents the most severe clinical presentation of peripheral arterial disease (PAD) in which the viability of tissues is threatened if arterial supply to the distal extremities is not timely restored. The Trans-Atlantic Inter-Society Consensus (TASC-II) [1] defines CLI as the presence of ischaemic rest pain or tissue lesions, such as non-healing wounds, necrosis or gangrene, which typically presents at the extremities of the affected limb for more than 2 weeks. This is usually associated with haemodynamic quantifications of ankle pressure <50 mmHg and toe pressure <30 mmHg in cases of ischaemic rest pain, or ankle pressure <70 mmHg and toe pressure <50 mmHg in cases of ischaemic ulcers or gangrene.

### Revascularisation

The main goals of revascularisation are to achieve reperfusion to the affected limb, to relieve ischaemic rest pain, heal chronic wounds, avert amputations, and maintain functional status of the patient [1, 2]. However, there is a dearth of robust evidence to inform clinical decisions [3, 4] in part because randomised controlled trials (RCTs) are ethically challenging to implement as treatment must be driven by patient-specific needs rather than research objectives.

Presently, the Bypass versus Angioplasty in Severe Ischaemia of the Leg (BASIL) trial [5] which ran from 1999 to 2004 remains the only RCT conducted to compare bypass interventions with plain balloon angioplasty in patients with CLI. However, there has since been a proliferation in endovascular technologies [6] and an improved understanding of the ameliorating factors in bypass surgeries [7], rendering the recommendations of the BASIL trial obsolete. Broad conclusions in CLI management have further been precluded by various impediments, such as heterogeneity in patient characteristics [8] and end-points in available studies [9], vague and controversial definitions of a non-salvageable limb [10], and considerable disparity in institutional protocols worldwide [11].

Amidst the ambiguity in revascularisation decisions, a consensus gained across international guidelines [1] and is firmly established in current practice is the targeting of the best vessel, or the least diseased artery supplying the best run-off to the foot. Yet, ischaemic ulcerations have been reported to persist and/or deteriorate despite technically successful revascularisations achieving the restoration of pedal pulses and vessel patency [12–15].

### The angiosome concept

**Fig. 1:**
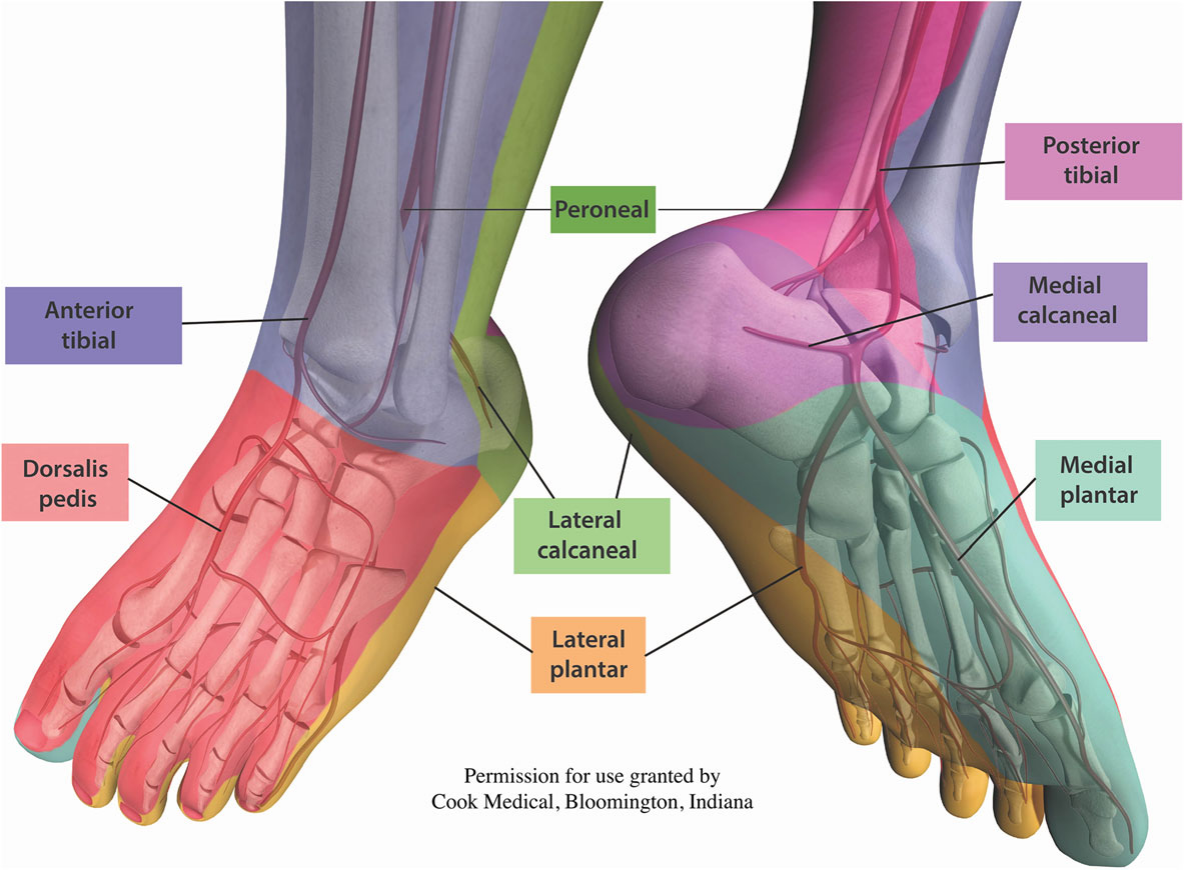
The Angiosome Concept

The angiosome concept (Fig. 1) [16], first proposed by Taylor and Palmer [17], was originally intended to provide a logical basis upon which to guide incisional strategies in plastic reconstructive surgery. It was later extrapolated to the management of CLI by Attinger and colleagues [18] in 2006. As it is beyond the scope of this review to thoroughly detail the concept, a summary of its key components is thus described.

Each angiosome is defined as a distinct anatomical territory, from cutaneous tissues through to bone, perfused by a single source artery. Six angiosomes have been demarcated in the foot and ankle which are supplied by three lower limb arteries–namely the Anterior Tibial, Posterior Tibial and Peroneal artery–and their respective branches. Smaller network of collaterals and arterial-arterial connections further interconnect each vascular territory, providing compensatory conduits of perfusion from adjacent angiosomes should vascular compromise in the source artery occur.

Utilising the angiosome concept in lower limb vascular surgery means a fundamental shift in the approach to the revascularisation strategy; with the choice of target vessel being guided by the site of ulceration rather than the least-diseased artery as identified on angiography [19]. This allows the ischaemic wound to be perfused by their respective source artery or ‘direct revascularisations’ (DR), instead of via collaterals or ‘indirect revascularisations’ (IR). Theoretically, clinical applications where this concept might be particularly relevant are in patient groups with notably compromised collaterals, such as those typically accompanying diabetes, renal insufficiency and in tobacco smokers.

While the angiosome concept remains a moot point in CLI surgery, as the concept was derived from selected healthy cadavers devoid of vascular compromise [17, 18], emerging evidence consisting of three meta-analyses [20–22] have cohesively indicated the clinical efficacy of DR over IR in relation to both wound healing and limb salvage outcomes. Although it is unclear if these benefits are applicable to a subgroup of patients with co-morbid diabetes [23], in whom the characteristics of PAD differ substantially from those without diabetes (Table 1), the results of a recently published meta-analysis [24] focusing solely on angioplasty interventions in this previously unexamined patient group had resonated with these findings.

**Table 1:** Comparison of PAD Characteristics [1, 23, 76–79]

	Patients with Diabetes	Patients without Diabetes
Age of onset	Younger	Older
Disease progression	Aggressive	Gradual
Anatomical localisation	• Mainly distal • Distinctly infrapopliteal affliction, frequently involving all three tibial region arteries: Anterior Tibial, Posterior Tibial and Peroneal artery • Relative sparing of inframalleolar pedal arteries (e.g. Dorsalis Pedis) and supragenicular arteries (e.g. Femoral and Aortic-iliac arteries).	• Mainly proximal • Lesions tend to affect the Femoral and Aortic-iliac arteries more frequently than the distal arteries
Type of atherosclerotic lesions	• Stenosis **<** Occlusions (severe) • Diffuse, and occurring over long segments	• Stenosis **>** Occlusions • Focal, and occurring over short segments
Calcification	Commonly present	Absent
Collateral network	Poor	Unaffected

### Diabetic population: unique challenges

The distinctive characteristics of PAD in patients with diabetes poses an added technical complexity to DR. Fundamentally, allowing the angiosome concept to modulate the revascularisation strategy in the diabetic population would mean having to recanalise a more calcified and occluded vessel over one which might be more pliable and patent.

### Clinical relevance

A focal point of the All-Party Parliamentary Group on Vascular Disease [25] is to promote ways to reduce avoidable lower limb amputations, especially those relating to diabetes and PAD. This is because while there is a complexity of factors contributing to non-healing diabetic foot ulcerations (DFU), PAD has been identified as the chief contributing factor [26]. Additionally, higher incidences of amputation despite technically successful revascularisations have been reported in certain patient subgroups. Patients with diabetes constitute one of those subgroups, for which a failure to reperfuse the site of tissue loss is identified as a leading factor [27].

### Statement of purpose

Hence, this literature review aims to examine the evidence to determine the comparative efficacy of a DR and IR strategy in optimising wound healing outcomes in patients with co-morbid diabetes and CLI with tissue loss.

## Methods

### Search strategy

A literature search was conducted in eight electronic databases, with the keywords determined after an initial browse on Google Scholar. Keywords, Medical Subject Headings (MeSH) terms and Boolean operators employed, along with further specifics of the search strategy, are detailed in Table 2. Listed in Additional file 1 is the database search record. The last search was conducted on 22 January 2017 and no time or language restrictions were set. The search strategy had been deliberately broad to capture all relevant literature.

**Table 2:** Literature Search Strategy

Search terms	S1–“critical limb isch?emia” OR “isch?emi*” S2–“peripheral arter* disease” OR “peripheral vascular disease” S3–“diabetic foot” OR “diabet*” S4–“bypass” OR “angioplasty” OR “endovascular” OR “revasculari?ation” OR “reconstruct*” S5–“angiosom*” OR “direct revasculari?ation” OR “indirect revasculari?ation” S6–S1 OR S2 OR S3 S7–S4 AND S5 AND S6
Databases searched	EBSCOhost (AMED, CINAHL), The Cochrane Library, ProQuest (ProQuest Health & Medicine Complete, ProQuest Nursing & Allied Health Source), PubMed, ScienceDirect, TRIP database
Part of journals searched	Title and Abstract
Years of search	No limits set
Language	No limits set

### Identification of studies

After removal of duplicates, articles were screened against the inclusion and exclusion criteria (Table 3) by their titles and abstracts. Articles which appeared eligible were then retrieved in full. Articles excluded at this stage were either inaccessible (Additional file 2) or found to be ineligible. The latter articles are listed in Additional file 3 together with the respective reasons for exclusion. Their reference lists were further examined for potential articles not retrieved in the electronic search; this process identified 15 additional articles.

**Table 3:** Inclusion and Exclusion Criteria

	Inclusion Criteria	Exclusion Criteria
Study design	• Full-text available in English • Cohort studies (retrospective/prospective)	• Non-English • Case reports, commentaries, reviews
Population (P)	• Human • Chronic limb ischaemia with tissue loss (Fontaine IV or Rutherford 5, 6) • Studies of interventions in patients with diabetes	• Cadaver or animal • Acute limb ischaemia • Chronic limb ischaemia without tissue loss (e.g. rest pain only; Fontaine III or Rutherford 4) • Mixed cohorts (i.e. not all patients have diabetes)
Intervention (I), Comparison (C)	• Arterial revascularisations • Revascularisation with application of the angiosome concept • Comparative study of DR and IR	• Non-arterial revascularisations • Revascularisation without application of the angiosome concept • Non-comparative studies of DR and IR
Outcome (O)	• Studies which utilised wound healing as an outcome measure	• Studies where wound healing was not utilised as an outcome measure

### Inclusion/exclusion criteria

#### Population (P)

Articles were restricted to cohorts comprised exclusively of patients with diabetes. Cohorts inclusive of patients with acute limb ischaemia were also intentionally excluded. As randomisations to equalise baseline confounders are ethically complex to achieve in surgical trials, including mixed cohorts will likely skew the results, leading to an overestimation of the true intervention’s effect.

#### Intervention (I)/comparison (C)

No exclusions were made on grounds of arterial interventional specifics due to the paucity of a clear evidence base. This was affirmed from the findings of a systematic review by the International Working Group on the Diabetic Foot (IWGDF) [28] and the TASC-II update published in 2015 [4] which found inconclusive evidence to further elucidate the revascularisation strategy. As it stands, diverse revascularisation techniques are utilised with dissimilar indications worldwide in the management of CLI [29]. Additionally, only comparative studies of DR and IR were included as this review aims to determine which approach is more efficacious.

#### Outcome (O)

Articles had to explicitly record wound healing as an outcome, as the persistence and deterioration of ischaemic ulcerations despite technically successful revascularisation hints that vessel patency, a physician-specific outcome, may not be a valid surrogate outcome measure. A decision was hence made to focus on wound healing as it is both a patient-centred and clinically meaningful outcome.

### Quality appraisal tools

Three methodological appraisal tools were considered, namely the Critical Appraisal Skills Programme [30] checklist, Newcastle-Ottawa Scale (NOS) [31] and Scottish Intercollegiate Guidelines Network [32] checklist. The NOS was ultimately chosen as not only was it recommended by The Cochrane Collaboration [33], it was also found to be the best available tool for assessing non-randomised studies [34]. Lastly, a publication from the IWGDF outlining the reporting standards for interventional studies in the management of DFUs [35] was utilised to underpin the quality appraisal of included studies.

## Results

**Fig. 2:**
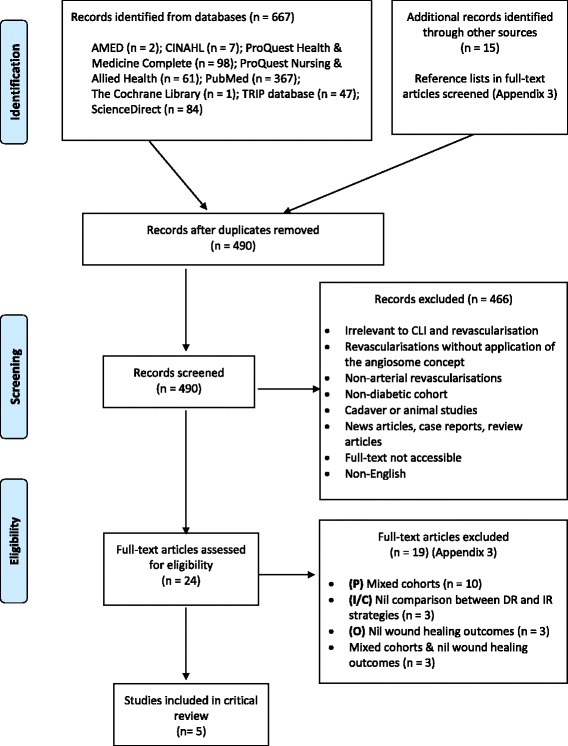
PRISMA Flow Diagram (adapted from [80])

Five studies [36–40] were ultimately enrolled for meeting the pre-established criterion. All five were published in peer-reviewed journals, are non-randomised, retrospective cohort studies and constitutes the highest level of contemporary evidence available to address the objective of this review. A PRISMA diagram delineating the search process is illustrated in Fig. 2. Key characteristics of each study are summarised in Table 4 and Additional file 4.

**Table 4:** Key Characteristics of Included Studies

	Fossaceca et al., 2013 [36]	Söderström et al., 2013 [37]	Acín et al., 2014 [38]	Lejay et al., 2014 [39]	Jeon et al., 2016 [40]
Participants	Italy, single-centre Retrospective, non-randomised Study period: 2005–2011 • 201 subjects (201 limbs) • Mean age: 75.5 (range 66–85) • PAD anatomical locations: Isolated BTK lesions • All foot ulcers	Finland, single-centre Retrospective, non-randomised Study period: 2007–2011 • 226 subjects (250 limbs) • Mean age: 71.1 (range 56.5–84.9) • PAD anatomical locations: Isolated infrapopliteal lesions • Ulcers distal to malleolus	Spain, single-centre Retrospective, non-randomised Study period: 1999–2009 • 92 subjects (101 limbs) • Mean age: 72 (range 64–77) • PAD anatomical locations: Femoropopliteal & infrapopliteal lesions • All foot ulcers	France, single-centre Retrospective, non-randomised Study period: 2003–2009 • 54 subjects (58 limbs) • Mean age: 69.1 (range 58–81) • PAD anatomical locations: Isolated BTK lesions • Ulcers distal to malleolus	South Korea, unspecified number of centres Retrospective, non-randomised Study period: 2011–2013 • 70 subjects (82 limbs) • Mean age: 69.6 (range 59.6–79.6) • PAD anatomical locations: Isolated infrapopliteal lesions • Ulcers distal to calcaneus
Diagnostic criterion for diabetes	Diagnostic criterion unstated in-text, however the following information was tabulated: • Time from diagnosis of diabetes: 12.5 ± 5.2 years • HbA1c (%): 7.9 ± 1.6 • Insulin therapy: 113 (56.2%)	• On hyperglycaemia reducing diet • Taking oral hypoglycaemic drugs • Undergoing insulin treatment	• Baseline blood glucose levels >120 g/dL, or • Require treatment with hypoglycaemic drugs	—	Diagnostic criterion unstated, however the following information was provided: • Mean duration of diabetes: 17.1 ± 9.7 years (range 1–50) • HbA1c (%): 8.5 ± 1.9
Intervention	Angioplasty: PTA • Primary endoluminal approach • Secondary subintimal approach	Angioplasty: PTA • Primary intraluminal approach	Stents used selectively Angioplasty: PTA • Primary endoluminal approach	Bypass • Autologous saphenous vein conduits only	Angioplasty: PTA • Primary intraluminal approach • Secondary subintimal approach
Guiding principle for interventions	Angiosome concept • i.e. all patients primarily considered for DR, subsequently undergoing IR when all DR options was not technically feasible	Best vessel strategy • i.e. retrospective grouping of patients into DR or IR, determined if the best quality vessel utilised supplied the ischaemic site via a source artery or via existing collaterals	Best vessel strategy • i.e. retrospective grouping of patients into DR or IR, determined if the best quality vessel utilised supplied the ischaemic site via a source artery or via existing collaterals	Angiosome concept • i.e. all patients primarily considered for DR, subsequently undergoing IR when DR was not technically feasible	Angiosome concept • i.e. all patients primarily considered for DR, subsequently undergoing IR when all DR options was not technically feasible
Pre-revascularisation care					
- Wound care	• Debridement of necrotic tissue	Local wound care tailored to lesion characteristics. • Debridement of devitalised tissue, surgical revision and indicated microbial therapy for infected ulcers; negative-pressure wound therapy and off-loading where indicated.	• Early debridement, abscess drainage, minor amputations, and wet dressings.	—	• Unstated.
- Medications	• Prophylaxis broad-spectrum antibiotic therapy • (The antibiotic utilised and the route of administration unstated.) • Dual anti-platelet therapy (Aspirin 100 mg/day, Clopidogrel 75 mg/day).	• Aspirin (100 mg/day), if not contraindicated.	• Broad-spectrum antibiotic therapy for severe infections in accordance with a general protocol. • (Protocol unstated, hence the drug utilised as well as the route of administration is not known.)	—	• Dual anti-platelet therapy at least 72 h before the procedure. (Aspirin 100 mg/day, Clopidogrel 75 mg/day)
Post-revascularisation care					
- Medications	• Dual anti-platelet therapy maintained (Aspirin 100 mg/day and Clopidogrel 75 mg/day) for 6 weeks, then Aspirin alone indefinitely.	• Lifelong Aspirin therapy, accompanied by Clopidogrel (75 mg/day) for 3 months after PTA	—	—	• Dual anti-platelet therapy maintained (Aspirin 100 mg/day and Clopidogrel 75 mg/day) once daily for at least 3 months if there were no contraindications to either drug.
Outcome measures					
- Wound healing	**✓**	**✓**	**✓**	**✓**	**✓**
	partial/complete at 1, 6, 12 months	at 12 months	at 12 months	at 3, 6, 12 months	at 12 months
- Limb salvage	**✓**	**✓**	**✓**	**✓**	**✓**
	at 1, 6, 12 months	at 12 months	at 24 months	at 12 months	at 12, 24 months
- Additional measures	Amputation (minor and major), Average TcPO_2_, Mortality, PTA retreatment, Restenosis, Technical success	AFS, AFS with healed ulcer, Median time to ulcer healing, Survival, Vascular Re-intervention	AFS, Major amputation at 30 days, MACE, MALE, Freedom from MALE + POD, Freedom from RAS, Freedom from RAO, Overall survival at 24 months	Median Ulcer Healing Time, Primary Patency, Survival, TcPO_2_	Amputation, Angiosome Score, Major and minor complications, Mortality, PTA reintervention, Technical Success, Wound Healing Time
Wound classification	—	UTWCS	—	UTWCS	Wagner
Presence of infection accounted for	—	**✓**	Graded according to CDC/NHSN surveillance definition [81]	**✓**	—
Follow-up (months)	• Protocol: 1, 6, 12 • Mean: 17.5 • Range 5.5–29.5	• Protocol: 1 month, and at 1–3 months thereafter depending on clinical condition of the foot • Mean: — • Range: — Surveillance of ulcer continued until healing occurred, with follow-up ending 1 year after intervention or death whichever occurred first.	• Protocol: 1, 3 and every 6 months thereafter. • Median: 19 • Range: 9–38	• Protocol: 1, 3, and every 6 months thereafter. • Mean: 20 • Range: 4–36	• Protocol: 12, 24 • Mean: 13 • Range: 0–25 The status of the wound was regularly checked until complete healing occurred.
Main findings: wound healing rate	No statistically significant difference found in therapeutic efficacy. (*p*-values: —)	• DR had a highly statistically significant improvement in wound healing rates at 12 months (*p* < 0.001) • Results were still highly statistically significant after adjustments with propensity score (HR 1.97; 95% CI, 1.34–2.90) (*p* = 0.001)	• DR had a highly statistically significant improvement in wound healing rates as compared to IR ‘without collaterals’ group at 12 months (*p* = 0.001) • No statistically significant differences were found between DR and IR ‘through collaterals’ groups for wound healing at 12 months (*p* = 0.38)	• DR had a statistically significant improvement in wound healing rates as compared to IR at 3, 6 and 12 months (*p* = 0.04)	• DR had a statistically significant improvement in wound healing rates as compared to IR at 12 months (*p* < 0.05)
Strengths of study	• TASC-II diagnostic criterion^a^ for CLI satisfied • Complete follow-up of all subjects • Diagnostic criteria of diabetes indicated • Subjects’ duration of diabetes provided	• TASC-II diagnostic criterion^a^ for CLI satisfied • Complete follow-up of all subjects • Diagnostic criteria of diabetes indicated • Consecutive sample • Employment of wound classification system • Presence of infection accounted for • Use of propensity score	• TASC-II diagnostic criterion^a^ for CLI satisfied • Diagnostic criteria of diabetes indicated • Consecutive sample • Presence of infection accounted for • Comparable baseline characteristics of subjects between groups	• TASC-II diagnostic criterion^a^ for CLI satisfied • Complete follow-up of all subjects • Consecutive sample • Employment of wound classification system • Presence of infection accounted for • Comparable baseline characteristics of subjects between groups	• TASC-II diagnostic criterion^a^ for CLI satisfied • Diagnostic criteria of diabetes indicated • Subjects’ duration of diabetes provided • Employment of wound classification system
Limitations of study	• Non-consecutive sample • Wound classification system not employed • Presence of infection not accounted for • Omission of subjects’ baseline characteristics	• No data on subjects’ duration of diabetes	• No data on subjects’ duration of diabetes • Drop-outs unaccounted • Wound classification system not employed • Patients with ESRD excluded	• No data on diagnostic criteria for diabetes • No data on subjects’ duration of diabetes	• Drop-outs unaccounted • Non-consecutive sample • Presence of infection not accounted for • Omission of subjects’ baseline characteristics
NOS scores	6/9	8/9	5/9	7/9	5/9

A tabulated summary of the key characteristics of included studies to allow easy visualisation and comparison across studies

*Abbreviations*: *AFS* Amputation-Free Survival, *ABPI* Ankle-Brachial Pressure Index, *BTK* Below-the-knee, *CDC* Centre for Disease Control and Prevention, *CLI* Critical Limb Ischaemia, *CI* Confidence Intervals, *DR* Direct Revascularisations, *DUS* Duplex Ultrasound, *ESRD* End-Stage Renal Disease, *HbA1c* Glycated haemoglobin, *HR* Hazard Ratio, *IR* Indirect Revascularisations, *MACE* Major adverse cardiovascular event, *MALE* Major adverse limb event, *NHSN* National Healthcare Safety Network, *NOS* Newcastle-Ottawa Scale, *TcPO*_*2*_ Transcutaneous oximetry, *PTA* Percutaneous Transluminal Angioplasty, *PAD* Peripheral Arterial Disease, *POD* Pre-operative Death, *RAO* Reintervention or amputation, *RAS* Reintervention, Amputation or Stenosis, *SPP* Skin Perfusion Pressure, *UTWCS* University of Texas Wound Classification System

Key: —, no data provided

^a^Additional details: TASC-II diagnostic criterion [1] is for the clinical diagnosis of CLI to be confirmed with objective quantifications of haemodynamic compromise, following the presence of symptoms for more than 2 weeks. The term CLI implies chronicity and is to be distinguished from acute limb ischemia

### Population characteristics

Whilst all studies comprised exclusively of subjects with diabetes, several details are noticeably absent. Evidently, all studies had omitted to distinguish the types of diabetes included and their relative proportions within the cohort. Lejay et al. [39] neglected to indicate their diagnostic criterion for diabetes, and three studies [37–39] neither specified the subjects’ duration of disease nor the adequacy of their glycaemic control. In addition, while the minimum reporting requirements for core patient details have been stated by the IWGDF [35] to be age, sex and ethnicity, all studies had omitted to document the ethnicity of their subjects. In terms of PAD lesion characteristics, while four studies comprised of subjects with isolated below-the-knee or infrapopliteal lesions, Acín and colleagues’ [38] study discordantly included subjects with femoropopliteal lesions. A breakdown of the baseline characteristics between DR and IR groups is tabulated in Table 5.

**Table 5:** Baseline Population Characteristics between DR and IR groups

	No. of patients	No. of limbs	Age	Male/Female	Ethnicity	HTN	DLP	History of smoking	ESRD/on dialysis	CAD	CVD
Fossaceca et al., 2013 [36]											
DR	201	-	75.5 ± 9.5	136 M/65 F	-	124 (62%)	-	-	15 (7%)	65 (32%)	-
IR											
*p*-values	NA	-	-	-	-	-	-	-	-	-	-
Söderström et al., 2013 [37]											
DR	226	121	68.4 ± 11.9	89 M/32 F	-	89 (74%)	78 (65%)	24 (18%)	26 (22%)	69 (57%)	29 (24%)
IR		129	73.8 ± 11.1	71 M/58 F	-	102 (79%)	84 (65%)	20 (24%)	13 (10%)	90 (70%)	24 (19%)
*p*-values	NA	NA	0.001	0.002	-	NS	NS	NS	0.012	0.044	NS
DR: propensity score matched pairs	-	84	71.7 ± 11.0	59 M/25 F	-	63 (75%)	50 (60%)	18 (25%)	14 (17%)	53 (63%)	20 (24%)
IR: propensity score matched pairs		84	70.3 ± 10.9	58 M/26 F	-	63 (75%)	60 (71%)	15 (20%)	12 (14%)	55 (66%)	17 (20%)
*p*-values: propensity score matched pairs	NA	NA	NS	NS	-	NS	NS	NS	NS	NS	NS
Acín et al., 2014 [38]											
DR	46	-	72 (63–78)	30 M/16 F	-	31 (67%)	13 (28%)	36 (78%)	Excluded	17 (37%)	9 (20%)
IR ‘through collaterals’	22	-	72 (68–75)	11 M/11 F	-	18 (82%)	9 (41%)	15 (68%)	Excluded	5 (23%)	6 (27%)
IR ‘without collaterals’	17	-	69 (63–77)	9 M/8 F	-	14 (82%)	4 (24%)	11 (65%)	Excluded	4 (24%)	3 (17%)
*p*-values: DR vs IR ‘through collaterals’	NA	-	NS	NS	-	NS	NS	NS	NS	NS	NS
*p*-values: DR vs IR ‘without collaterals’	NA	-	NS	NS	-	NS	NS	NS	NS	NS	NS
Lejay et al., 2014 [39]											
DR	36	-	68 ± 10	25 M/11 F	-	34 (95%)	19 (53%)	25 (69%)	19 (53%)	19 (53%)	4 (11%)
IR	22	-	71 ± 10	15 M/7 F	-	21 (96%)	12 (55%)	16 (73%)	12 (55%)	12 (55%)	2 (9%)
*p*-values	NA	-	NS	NS	-	NS	NS	NS	NS	NS	NS
Jeon et al., 2016 [40]											
DR	70	63	69.6 ± 10	51 M/19 F	-	63 (90%)	-	-	24 (34%)	31 (44%)	-
IR		19									
*p*-values	NA	NA	-	-	-	-	-	-	-	-	-

A detailed breakdown of baseline population characteristics as derived from primary studies

*Abbreviations*: *CAD* Coronary Artery Disease, *CVD* Cerebrovascular Disease, *ESRD* End-Stage Renal Disease, *DLP* Dyslipidaemia, *HTN* Hypertension, *NA* Not applicable, *NS* Not significant; where *p* ≥ 0.05

Key: —, no data provided

### Intervention

Across all studies, variations are apparent in arterial interventional specifics (Table 4). Revascularisation interventions are also noted to be fundamentally guided by two differing strategies. In two studies [37, 38], revascularisations were principally guided by the best vessel strategy. With this strategy, patients were retrospectively grouped into DR or IR depending on whether the vessel utilised had reperfused the ischaemic ulcer via a source artery or collaterals. In the remaining three studies [36, 39, 40], revascularisations were guided by the angiosome concept. Following this strategy, the wound related artery was initially targeted in all patients. The best available vessel was subsequently recanalised after all DR options could not be achieved.

### Outcome measures

While differences are evident in follow-up protocols, wound healing at 12 months is noted to be the only outcome uniformly measured and consistently defined. All studies defined it as complete epithelialisation, a definition congruent with IWGDF recommendations [41]. Wounds were considered non-healing should full epithelisation either not occur within the specified follow-up timeframe or where amputation was necessitated.

Ulcerations in all subjects were found to be localised to the foot. Notably, only two studies [39, 40] had specified ulcer duration and three studies [37, 39, 40] had classified the anatomical depth of ulcerations. Two papers [37, 39] had utilised the University of Texas Wound Classification System, whereas Jeon and colleagues [40] utilised the Wagner classification system. Additionally, all but two studies [36, 40] had noted the presence of infection, with subjects affected categorically analysed in further subgroups.

All studies also homogenously defined limb salvage as the avoidance of amputation proximal to the ankle joint. However, while four studies had utilised 12 months as an end-point, Acín et al.’s [38] study incongruously utilised a 24-month end-point with no information provided in the prior period, limiting efforts in drawing comparisons.

## Analysis

### Population characteristics

Although the omission of certain attributes within studies is insufficient as to invalidate their results, the reliability of their findings is threatened. Firstly, correlations were found between duration of diabetes [42] and HbA1c levels [43] with PAD severity, which could act as unmeasured confounders in three studies [37–39] who had neglected to report this detail. Secondly, there is evidence suggesting dissimilar anatomical patterns of PAD between ethnic groups [44–47] which could present another unmeasured confounder across studies. Nevertheless, PAD lesion characteristics of subjects appear to be broadly similar, allowing for sound comparisons. Thirdly, although all studies did not specify the types of diabetes included and their relative proportions within the cohort, this appears not to be a confounding factor as no discernible differences were found in the micro- and macrovascular comorbidities between patients with type 1 and type 2 diabetes [48]. Lastly, relating solely to Acín et al. [38]’s study, it is equivocal as to how the inclusion of subjects with femoropopliteal lesions had influenced their findings. The reason being while a French study [49] of 400 non-consecutive PAD patients had found proximal-level PAD to be independently associated with a poorer prognosis, a later American study [50] of 12,731 consecutive PAD patients contradictorily found distal-level PAD to have this association.

### Intervention

Inconsistencies in arterial interventional specifics are evident across all studies as essential components of an optimal revascularisation strategy remain indeterminate. As such, even though heterogeneity in this regard is a tenable but recognised limitation which impinges on the internal validity of this review, it is reflective of current practice and retains good external validity.

Regarding the discrepancy noted in fundamental principles guiding revascularisations, it is unknown if this difference is consequential. This is because it remains undetermined if the quality of conduit or target of vessel outflow is a greater determinant of intervention outcomes in the diabetic population. Arguably, given that Lejay et al.’s [39] study utilised bypass interventions with autologous saphenous vein conduits only, all of their subjects can be deemed to be recanalised with the best quality conduit as atherosclerosis primarily affects arteries and not veins.

### Outcome measures

All ulcerations are pertinently localised to the foot as inframalleolar ulcerations are more likely to be arterial in aetiology; in contrast, supramalleolar ulcerations are predominantly venous-related [1]. However, even though three studies [37, 39, 40] had utilised well-established classification systems, the Wagner classification have not been externally validated [51] such that its refrained use in DFU assessments is clearly expressed in the National Institute for Health and Care Excellence guidelines [52]. Moreover, the lack of distinction between subjects with and those without infection undermines the results of two studies [36, 40] as infection is a considerable aggravating factor [53] hampering wound-healing ability.

Pertaining solely to Lejay et al.’s [39] study, it must be highlighted their assessment of wound healing was rescinded should an ulcer recur within 3 months of complete epithelisation. On one hand, this increases the robustness of their findings as DFU recurrence rates are high with reported rates of 40% within the first year [54]. On the other hand, it casts an undeterminable degree of ambiguity over their findings as a multiplicity of factors can lead to wound recalcitration. Predictive factors, such as type and severity of foot deformity, degree of peripheral neuropathy and positive history of ulceration [55] have not been accounted for and no mention was made on the number of subjects affected by this stipulation.

### Completeness of follow-up

A striking methodological flaw specific to two studies [38, 40] is even though they had noted a 10.8 and 15.5% attrition rate respectively, no comparative analysis was subsequently made between subjects lost to follow-up and those followed in full. Consequently, their findings ought to be interpreted cautiously as even minimal losses can introduce bias should the reasons for loss be related to outcome status.

### Methodological rigour

Each study was critically appraised using the NOS, and all studies scored between 5 and 8 out of a maximum score of 9. The scoring process is detailed in Additional file 5. As the threshold scores for distinguishing between methodologically ‘good’ and ‘poor’ studies have not been established [31], studies which scored 5 and 6 will herein be considered ‘methodologically weak’, while the studies which scored 7 and 8 will be considered ‘methodologically strong’. Across all five studies, results were taken to be statistically significant when *p* < 0.05.

Whilst it is not possible to disregard the drawbacks inherent in a retrospective study design, some studies had taken appropriate measures to minimise the influence of these elements. Firstly, three cohorts [37–39] comprised of a consecutive sample of subjects, effectively upholding the impartiality of their data. Having lapsed in this regard, it is difficult to ascertain the neutrality of data in the remaining two studies [36, 40] from selection bias. A further flaw discrediting their findings is the failure to detail the groups’ baseline characteristics. The omission of this key attribute cast doubts on their rigour as ill-matched comparative groups can have direct bearings on results.

Secondly, due to legitimate ethical constraints, none of the papers had employed the methods of blinding or randomisation to achieve an unbiased apportion of confounders. Exceptionally, only Söderström et al. [37] had utilised a propensity score to adjust for overt baseline disparities. This strengthens the validity of their findings as differences in results can be more confidently attributed to the factor under investigation. Nevertheless, baseline characteristics of subjects between DR and IR groups in Acín et al. [38] and Lejay et al.’s [39] studies were notably uniform and statistically non-significant (Table 5). On this account, comparability can reasonably be conceded, giving credence to their findings.

Notwithstanding the drawbacks mentioned, a strength inherent to all five studies relates to their non-experimental study designs. With broader inclusion criteria than experimental controlled trials, their study cohorts are more likely to be representative of the diverse patient populations seen in practice. Additionally, their studies reflect real-world treatment decisions and management protocols, producing results with greater generalisability [56]. Notably, Acín et al. [38] is the only study poorly representative of the diabetic population. This is because diabetes is the leading cause of end-stage renal disease and an estimated 50% of these patients have diabetes [57], yet this subgroup of patients was excluded.

### Primary outcome measure: wound healing rates

Findings pertaining to wound healing outcomes at 12 months were incongruous, likely resulting from their varying methodological designs. The numerous methodological flaws in three studies [36, 38, 40] leave their results vulnerable to type I and type II errors, making it injudicious to attribute weight to their findings.

Focusing on methodologically stronger studies [37, 39], giving an aggregated sample of 280 subjects, statistically significant improvements for wound healing via DR were found with *p*-values of <0.001 and 0.04 respectively, signifying the unlikelihood for differences between interventions to have arisen by chance. This is further affirmed by Söderström and associates’ [37] study who indicated a nearly twofold increased probability (hazard ratio, 1.97) for subjects undergoing DR to achieve wound healing in 12 months. While the aforementioned findings concur in the clinical superiority of DR, the substantial interval at a 95% confidence level (95% CI, 1.34–2.90) reveals the considerable uncertainty inherent in the researchers’ estimate of the probability of increased benefit afforded by DR over IR. Potential reasons include their small cohort size (*n* = 226), making it difficult to extrapolate their findings to the entire diabetic population, and heterogeneity in subjects’ covariates unmatched by the propensity score, for instance, glycaemic control and ulceration characteristics. These findings denote that achieving direct arterial perfusion to the site of ulceration may be important for patients with diabetes. Incorporating the angiosome concept as an adjuvant consideration to the best vessel revascularisation strategy could therefore present a potential to optimise wound healing outcomes in patients with co-morbid diabetes and CLI.

## Discussion

Over 80% of diabetes-related amputations are preceded by a non-healing foot ulcer [52], presenting a considerable economic challenge and demand on healthcare systems worldwide. After careful and rigorous scrutiny of contemporary evidence, DR appears to be more efficacious than IR in optimising wound healing outcomes and may contribute better towards the global endeavour of reducing avoidable non-traumatic lower limb amputations in patients with diabetes [25, 58, 59].

These findings raise the possibility that the unaccounted relationship between the target vessel and the site of ulceration might be part of the reason why ischaemic ulcerations persist and/or deteriorate despite technically successful revascularisations. However, as the current evidence-base is still finite and of limited quality, no definitive recommendations can be drawn from this review. Further investigations are warranted to evidence the impact of incorporating a DR approach within the conventional revascularisation strategy on the healing rates of chronic DFUs. Further investigations are also necessary to reconcile the contradictory findings of studies supporting the clinical superiority of DR over IR [37–40, 60–62] with those which had found no significant difference in strategies [36, 63–67] for this subgroup.

### Implications for practice

While the criterion for a diagnosis of CLI is clear, the subsequent management of patients with CLI is fraught with innumerable complexities. The decision regarding interventional specifics, and principally to undergo or forgo revascularisation, is complex and requires deliberation with all key stakeholders. This is because treatment decisions should be based not only on local availability of facilities and skills, but should also accord due respect to the patient’s preferences [1, 68]. Crucially, it must further be recognised that amputation or the continuation of conservative treatments can be favourable and therapeutic options in patients who are unlikely to benefit from revascularisation [69, 70]. As it is not clear at present how practice guidelines should change to accommodate these considerations, it is important for vascular surgeons, specialist podiatrists and key personnel involved in the management of DFUs to be keenly aware of the dynamic evidence-base underpinning different procedural types, for this will provide a sound basis for their provision of individually tailored treatments.

### Multidisciplinary implications

**Fig. 3:**
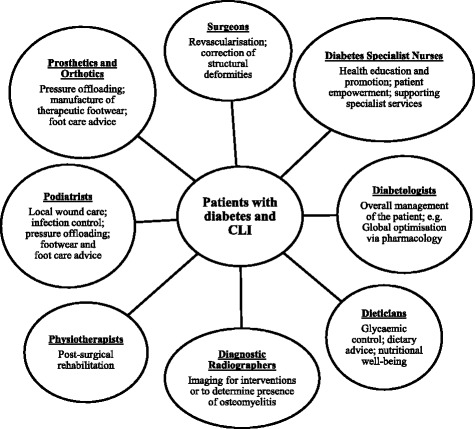
Multidisciplinary Approach for global patient management [82–84]

While acknowledging PAD as the predominant factor contributing to non-healing DFUs, it must be recognised it is but one of a multitude of factors impairing wound healing ability. Contributory local components, which may include but is not limited to, disproportionate plantar pressure distribution, severity of peripheral neuropathy and polymicrobial infection [71] must all be effectively mitigated for ulcer resolution. Critically, practitioners must remain cognizant that patients with CLI and co-morbid diabetes are also afflicted with severe cardiovascular comorbidities. Mortality rates are dire–with approximately 50% of people dying within 5 years of presenting with a DFU and up to 70% of people dying 5-years post-amputation [52]–reflecting the medical acuity of these patients. Given that numerous risk factors contributing towards cardiovascular disease can be negated with lifestyle modification, efforts in health promotion toward key areas such as smoking cessation and optimal management of the triad of hypertension, hyperlipidaemia and hyperglycaemia provide opportunities to not only improve intervention outcomes but also maintain systemic well-being. Consequently, it is of paramount importance to adopt a comprehensive and well-integrated multidisciplinary approach (Fig. 3) for successful global patient management.

### Limitations

The findings of this review must be interpreted in light of its limitations. Firstly, the inaccessibility to numerous articles (Additional file 2), including articles not published in English, prohibited the inclusion of further studies. Secondly, all studies are retrospective in nature hence the potential for residual confounding in outcomes by unmeasured variables such as anatomical variability, quality of existing collaterals and the pedal arch cannot be excluded. Lastly, while the limited internal validity in this review have been acknowledged, looking to the future, three multi-centre RCTs are underway and is anticipated to elucidate the interventional specifics of CLI revascularisation. They are the BASIL-2 [72], BAlloon versus Stenting in Severe Ischaemia of the Leg-3 (BASIL-3) [73] and the Best Endovascular versus Best Surgical Therapy in Patients with CLI (BEST-CLI) Trial [74].

### Recommendations for future research

With the limited data currently available, findings appear promising and merit from further investigations, particularly to ascertain the degree of comparative efficacy afforded by DR over IR in the diabetic population. It is imperative to rigorously assess and substantiate the short- and long-term safety and viability of incorporating a DR approach in methodologically robust and adequately powered prospective studies before any revisions to the conventional revascularisation strategy can be justified. Future research efforts are recommended to comply with the European Wound Management Association’s recommendations [53, 75] to ensure consistency in outcome measurements and to pay heed to the reporting standards outlined by IWGDF [35] to improve the quality of their studies.

## Conclusion

As the evidence-base is of limited quality and quantity, no definitive recommendations can be drawn from this review. However, with the finite data available, it appears recalibrating the conventional revascularisation strategy to incorporate the angiosome concept may be more efficacious than a solely indirect approach in optimising wound healing outcomes for patients with co-morbid diabetes and CLI.

## Additional files

Additional file 1: Database Search Record. (DOCX 17 kb)

Additional file 2: Only Abstracts Accessible. (DOCX 19 kb)

Additional file 3: Full-text Articles Excluded. (DOCX 21 kb)

Additional file 4: Literature Review Tables. (DOCX 34 kb)

Additional file 5: The Newcastle-Ottawa Scale (NOS) scores. (DOCX 20 kb)

## Abbreviations

BASIL: Bypass versus Angioplasty in Severe Ischaemia of the Leg
CLI: Critical limb ischaemia
DFU: Diabetic foot ulceration
DR: Direct revascularisations
IR: Indirect revascularisations
IWGDF: International Working Group on the Diabetic Foot
NOS: Newcastle-Ottawa Scale
PAD: Peripheral arterial disease
RCT: Randomised controlled trial
TASC: Trans-Atlantic Inter-Society Consensus
