# Supplementary material for: The comparative efficacy of angiosome-directed and indirect revascularisation strategies to aid healing of chronic foot wounds in patients with co-morbid diabetes mellitus and critical limb ischaemia: a literature review
Source: J Foot Ankle Res. 2017 Jun 28;10:26. doi: 10.1186/s13047-017-0206-5 (PMC5490238; doi:10.1186/s13047-017-0206-5)
Supplement: Supplementary file 1 — Database Search Record. (DOCX 17 kb) [file 13047_2017_206_MOESM1_ESM.docx]

**Additional file 1: Database Search Record**

| **No.** | **Database** | **Keywords** | **Hits** |
| --- | --- | --- | --- |
| **1** | EBSCOhost (AMED) | **S1** - “critical limb isch?emia”  **S2** - “isch?emi*”  **S3** - “peripheral arter* disease”  **S4** - “peripheral vascular disease”  **S5** - “diabetic foot”  **S6** - “diabet*”  **S7** - S1 OR S2 OR S3 OR S4 OR S5 OR S6  **S8** - “bypass”  **S9** - “angioplasty”  **S10** - “endovascular”  **S11** - “revasculari?ation”  **S12** - “reconstruct*”  **S13** – S8 OR S9 OR S10 OR S11 OR S12  **S14** - “angiosom*”  **S15** - “direct revasculari?ation”  **S16** - “indirect revasculari?ation”  **S17** - S14 OR S15 OR S16  **S18** - S7 AND S13 AND S17 | 2 |
| **2** | EBSCOhost (CINAHL) | **S1** - “critical limb isch?emia”  **S2** - “isch?emi*”  **S3** - (MH “Peripheral Vascular Diseases”)  **S4** - “peripheral arter* disease”  **S5** – (MH “Diabetic Foot”)  **S6** - (MH “Diabetes Mellitus”) OR “diabet*”  **S7** - S1 OR S2 OR S3 OR S4 OR S5 OR S6  **S8** - “bypass”  **S9** – (MH “Angioplasty”)  **S10** - “endovascular”  **S11** – (MH “Revascularization“) OR “revasculari?ation”  **S12** – (MH “Surgery, Reconstructive”) OR “reconstruct*”  **S13** – S8 OR S9 OR S10 OR S11 OR S12  **S14** - “angiosom*”  **S15** - “direct revasculari?ation”  **S16** - “indirect revasculari?ation”  **S17** - S14 OR S15 OR S16  **S18** - S7 AND S13 AND S17 | 7 |
| 3 | ProQuest (ProQuest Health & Medical Complete) | (Critical limb isch?emia) OR isch?emi* OR (peripheral arter* disease) OR (peripheral vascular disease) OR (diabetic foot) OR diabet*  **AND**  Bypass OR angioplasty OR endovascular OR revasculari?ation OR reconstruct*  **AND**  (angiosom*) OR (direct revasculari?ation) OR (indirect revasculari?ation) | 98 |
| 4 | ProQuest (Nursing & Allied Health Source) | (Critical limb isch?emia) OR isch?emi* OR (peripheral arter* disease) OR (peripheral vascular disease) OR (diabetic foot) OR diabet*  **AND**  Bypass OR angioplasty OR endovascular OR revasculari?ation OR reconstruct*  **AND**  (angiosom*) OR (direct revasculari?ation) OR (indirect revasculari?ation) | 61 |
| 5 | PubMed | Search ischaemi*  Search peripheral arter* disease  Search Peripheral Vascular Disease  Search Peripheral Arterial Disease  Search Peripheral Arter* Disease  Search Diabetic Foot  Search diabet*  Search ((((((critical limb ischaemia) OR ischaemi*) OR peripheral arter* disease) OR Peripheral Vascular Disease) OR Peripheral Arterial Disease) OR Diabetic Foot) OR diabet*  Search bypass  Search Angioplasty  Search Endovascular Procedures  Search revascularisation  Search revascularization  Search critical limb ischaemia  Search Reconstructive Surgical Procedures  Search reconstruct*  Search ((((((bypass) OR Angioplasty) OR Endovascular Procedures) OR revascularisation) OR revascularization) OR Reconstructive Surgical Procedures) OR reconstruct*  Search angiosom*  Search direct revascularisation  Search direct revascularization  Search indirect revascularisation  Search indirect revascularization  Search ((((angiosom*) OR direct revascularisation) OR direct revascularization) OR indirect revascularisation) OR indirect revascularization  Search (((((((((critical limb ischaemia) OR ischaemi*) OR peripheral arter* disease) OR Peripheral Vascular Disease) OR Peripheral Arterial Disease) OR Diabetic Foot) OR diabet*)) AND (((((((bypass) OR Angioplasty) OR Endovascular Procedures) OR revascularisation) OR revascularization) OR Reconstructive Surgical Procedures) OR reconstruct*)) AND (((((angiosom*) OR direct revascularisation) OR direct revascularization) OR indirect revascularisation) OR indirect revascularization) | 367 |
| 6 | The Cochrane Library | (Critical limb isch?emia) OR isch?emi* OR (peripheral arter* disease) OR (peripheral vascular disease) OR (diabetic foot) OR diabet*  **AND**  Bypass OR angioplasty OR endovascular OR revasculari?ation OR reconstruct*  **AND**  (angiosom*) OR (direct revasculari?ation) OR (indirect revasculari?ation) | 1 |
| 7 | TRIP database | Angiosom* | 47 |
| 8 | ScienceDirect | (Critical limb isch?emia) OR isch?emi* OR (peripheral arter* disease) OR (peripheral vascular disease) OR (diabetic foot) OR diabet* OR bypass OR angioplasty OR endovascular OR revasculari?ation OR reconstruct*  **AND**  angiosom* | 84 |
