# Supplementary material for: The comparative efficacy of angiosome-directed and indirect revascularisation strategies to aid healing of chronic foot wounds in patients with co-morbid diabetes mellitus and critical limb ischaemia: a literature review
Source: J Foot Ankle Res. 2017 Jun 28;10:26. doi: 10.1186/s13047-017-0206-5 (PMC5490238; doi:10.1186/s13047-017-0206-5)
Supplement: Supplementary file 2 — Only Abstracts Accessible. (DOCX 19 kb) [file 13047_2017_206_MOESM2_ESM.docx]

# Additional file 2: Only Abstracts Accessible

| **No.** | Citation | Country |
| --- | --- | --- |
| 1 | Alexandrescu VA, Hubermont G, Philips Y, Guillaume B, Ngongang C, Vandenbossche P, et al. Primary Angioplasty Guided By An Angiosome Model Of Reperfusion In The Treatment Of Ischemic Inferior Limb Diabetic Wounds: Preliminary Results. *The American Journal of Cardiology.* 2009;104(Suppl 6):214D. Available from: doi: 10.1016/j.amjcard.2009.08.615 [Accessed 16 January 2016] | Belgium |
| 2 | Blanes Ortí P, Riera Vázquez R, Puigmacià Minguell R, Valverde García S, Manuel­Rimbau Muñoz E, Lozano Vilardell P. Revascularización percutánea de angiosomas específicos en isquemia crítica de la extremidad. *Angiología.* 2011;63(1):11­17. Available from: doi: 10.1016/S0003-3170(11)70063-3 [Accessed 24 January 2017] | Spain |
| 3 | Coppelli A, Lacopi E, Bargellini I, Cicorelli A, Lunardi A, Mattaliano C, et al. E*ndovascular revascularisation in type 2 diabetic patients with critical limb ischaemia:comparison of direct and indirect revascularisarion according to angiosome model. 2014.* Available from: http://www.easdvirtualmeeting.org/resources/endovascular-revascularisation-in-type-2-diabetic-patients-with-critical-limb-ischaemia-comparison-of-direct-and-indirect-revascularisarion-according-to-angiosome-model--2 [Accessed 27 January 2017] | Austria |
| 4 | Coppelli A, Lacopi E, Bargellini I, Goretti C, Cicorelli A, Lunardi A, et al. Direct Revascularization Based on the Angiosome Model (AM) Reduces Risk of Major Amputations and Increases Life Expectancy in Diabetic Patients with Critical Limb Ischemia (CLI) and Diabetic Foot Ulceration (DFU). *Diabetes.* 2015;64(Suppl 1):A36. Available from: https://www.researchgate.net/publication/296088559_Direct_Revascularization_Based_on_the_Angiosome_Model_AM_Reduces_Risk_of_Major_Amputations_and_Increases_Life_Expectancy_in_Diabetic_Patients_with_Critical_Limb_Ischemia_CLI_and_Diabetic_Foot_Ulcerati [Accessed 27 January 2017] | Italy |
| 5 | Deguchi J, Kitaoka T, Yamamoto K, Matsumoto H, Sato O. Impact of angiosome on treatment of diabetic ischaemic foot with paramalleolar bypass. *The Journal of Japanese College of Angiology*. 2010;50:687–691. | Japan |
| 6 | Ferrufino-Mérida AL, Rodríguez-Trejo JM, Escotto-Sánchez I, Rodríguez-Ramírez N. Angioplastia infrapoplítea: correlación entre el vaso tratado y el angiosoma lesionado. *Revista Mexicana de Angiología*. 2012;40(4):123-134. Available from: http://new.medigraphic.com/cgi-bin/resumen.cgi?IDARTICULO=43199 [Accessed 27 January 2017] | Spain |
| 7 | Gargiulo M, Schneider FM, Stella A, Abualhin M, Desvergnes M, Ricco JB. Utility of Direct Angiosome Revascularization and Runoff Scores in Predicting Limb Salvage in Patients Undergoing Peroneal Bypass for Tissue Loss: A Propensity Analysis. *Journal of Vascular Surgery*. 2015;61(6S):37S-38S. Available from: doi: 10.1016/j.jvs.2015.04.066 [Accessed 16 January 2016] | Italy |
| 8 | Glasbey JCD, Bosanquet DC, Williams IM, Twine CP. Is direct angiosomal revascularisation superior to indirect revascularisation for infrapopliteal arterial disease?. *International Journal of Surgery.* 2014;12(3):S115-S116. Available from: doi: 10.1016/j.ijsu.2014.08.335 [Accessed 24 January 2017] | UK |
| 9 | Goatman C, Antoniou GA, Antoniou SA, Kuhan G, Georgiadis GS, Murray D. The angiosome model as an effective paradigm to improve clinical outcomes of infra-popliteal revascularization. *International Journal of Surgery*. 2013;11(8):739. Available from: doi: 10.1016/j.ijsu.2013.06.813. [Accessed 24 January 2017] | UK |
| 10 | Harth C, Randon C, Vermassen F. Impact of Angiosome Targeted Femorodistal Bypass Surgery on Healing Rate and Outcome in Critical Limb Ischemia. *European Journal of Vascular and Endovascular Surgery.* 2015;50(3):398-399. Available from: doi: 10.1016/j.ejvs.2015.06.055 [Accessed 16 January 2016] | Belgium |
| 11 | Kaputin MLU, Platonov SA, Ovcharenko DV, Voronkov AA, Kiselev MA. Angiographic characteristics of the lesion, influencing the choice to perform either direct or indirect endovascular revascularization for critical ischaemia of the lower limbs. *Angiologiya i Sosudistaya Khirurgiya*. 2013;19(1):47-51. Available from: https://www.ncbi.nlm.nih.gov/pubmed/23531659. [Accessed 22 January 2017] | Russia |
| 12 | Lacopi E, Coppelli A, Bargellini I, Cicorelli A, Lunardi A, Mattaliano C. Clinical outcomes after endovascular revascularization in type 2 diabetic patients with critical limb ischemia: Comparison of direct and indirect revascularization according to the angiosome model. *EWMA*. 2014:9. Available from: http://old.ewma.org/fileadmin/user_upload/EWMA/pdf/conference_abstracts/2014/Oral/Abstracts_ENG_til_Web_Part9.pdf [Accessed 27 January 2017] | Italy |
| 13 | Lee CH, Kwon O, Do U, Hong JA, Lee K, Cho MS, et al. Angiosome-target single-vessel angioplasty is not inferior to multi-vessel angioplasty in patients. *Journal of the American College of Cardiology.* 2016;68(18 Suppl 1):B316-B317. Available from: doi: 10.1016/j.jacc.2016.09.814 [Accessed 24 January 2017] | Korea |
| 14 | Lida O, Uematsu M, Soga Y, Suzuki K, Yokoi H, Nobuyoshi M, et al. Impact of angiosome in endovascular therapy on the limb salvage for the patients with critical limb schema presenting with isolated infrapopliteal lesions. *Journal of the American College of Cardiology.* 2011;57(14 Suppl 1):E1667. Available from: doi: 10.1016/S0735-1097(11)61667-7 [Accessed 16 January 2016] | Japan |
| 15 | Nanto K, Lida O, Okamoto S, Dohi T, Soga Y, Hirano K, et al. Impact of Angiosome Approach on Clinical Outcomes of Endovascular Therapy in Patients with Critical Limb Ischemia Presenting with Isolated Below-the- Knee Lesions. *Journal of the American College of Cardiology.* 2011;58(20 Suppl B):B151. Available from: doi: 10.1016/j.jacc.2011.10.573 [Accessed 16 January 2016] | Japan |
| 16 | Nishijima T, Ono T, Morihisa K, Kugimiya F, Fukushima H, Sumida H, et al. Direct flow utilizing the angiosome concept is valuable for salvaging limbs in critical limb ischemia patients. *European Heart Journal.* 2013;34(Suppl 1):P375. Available from: doi: 10.1093/eurheartj/eht307.P375 [Accessed 27 January 2017] | Japan |
| 17 | Osawa S, Terashi H, Tsuji Y, Kitano I, Sugimoto K. Importance of the six angiosomes concept through arterial-arterial connections in CLI. *International Angiology.* 2013;32(4):375-385. Available from: http://www.ncbi.nlm.nih.gov/pubmed/23822940 [Accessed 16 January 2016] | Japan |
| 18 | Oshima S, Noda K, Sumida H, Nishijima T, Siratake Y. Impact of the angiosome concept for endovascular therapy in patients with critical limb ischemia due to isolated below-the knee lesions. *The Journal of Japanese Society of Limb Salvage and Podiatric Medicine.* 2013;5(2):85-90. Available from: https://www.jstage.jst.go.jp/article/jlspm/5/2/5_85/_article [Accessed 16 January 2016] | Japan |
| 19 | Sharrock AE, Barker T, Kershaw EL, Watts C. Keeping an Eye on the Target: Direct or Indirect Revascularisation of the Angiosome?. *European Journal of Vascular & Endovascular Surgery.* 2015;50(3):216. Available from: doi: 10.1016/j.ejvs.2015.06.013 [Accessed 16 January 2016] | [UK](http://dx.doi.org/10.1016/j.ejvs.2015.06.013) |
| 20 | Slim H, Zayed H, Edmonds M, Rashid H. The Impact of Arterial Pedal Arch Quality and Angiosome Revascularization on the Outcome of Distal Bypass Surgery. *Journal of Vascular Surgery.* 2012;55(Suppl 6):16S-17S. Available from: doi: 10.1016/j.jvs.2012.03.067 [Accessed 16 January 2016] | [UK](http://dx.doi.org/10.1016/j.ejvs.2015.06.013) |
| 21 | Soon C, Tay K, Taneja M, Teo T, Lo R, Burgmans MC et al. Angiosome directed angioplasty for limb salvage in critical limb ischemia. *Journal of Vascular and Interventional Radiology.* 2012;23(Suppl 3):S57. Available from: doi: 10.1016/j.jvir.2011.12.180 [Accessed 16 January 2016] | [Singapore](http://dx.doi.org/10.1016/j.ejvs.2015.06.013) |
| 22 | Špillerová K, Biancari F, Leppäniemi A, Albäck A, Söderström M, Venermo M. The Importance of Angiosome Concept on Ulcer Healing: Percutaneous Transluminal Angioplasty vs. Surgical Bypass in Bellow the Knee Arteries. *European Journal of Vascular and Endovascular Surgery.* 2014;48(3):341-342. Available from: doi: 10.1016/j.ejvs.2014.06.012 [Accessed 16 January 2016] | [Finland](http://dx.doi.org/10.1016/j.ejvs.2015.06.013) |
| 23 | Špillerová K, Biancari F, Albäck A, Venermo M. Prognostic Significance of Different Strategies of Angiosome Targeted Lower Limb Revascularization*. European Journal of Vascular and Endovascular Surgery*. 2015;50(3):399. Available from: doi: 10.1016/j.ejvs.2015.06.057 [Accessed 16 January 2016] | [Finland](http://dx.doi.org/10.1016/j.ejvs.2015.06.013) |
| 24 | Stempfle HU, Remp T, Kulzer M, Kreider M. Endovascular infrapopliteal interventions guided by an angiosome-based concept will improve clinical outcome in critical limb ischemia. *European Heart Journal*. 2014;35(Suppl):631. | [Germany](http://dx.doi.org/10.1016/j.ejvs.2015.06.013) |
| 25 | Stimpson AL, Hanif U, Germain S, Chick C, Goyal N, Twine CP. Combined Infra-popliteal Angioplasty has No Advantage over Direct Angiosomal Revascularisation. *European Journal of Vascular and Endovascular Surgery.* 2015;50(3):399-400. Available from: doi: 10.1016/j.ejvs.2015.06.058 [Accessed 16 January 2016] | [Belgium](http://dx.doi.org/10.1016/j.ejvs.2015.06.013) |
| 26 | Yie K. Angiosome Guided Surgical Revascularization; the Truth and Falsehood. *European Journal of Vascular & Endovascular Surgery.* 2014;48(3):346. Available from: doi: 10.1016/j.ejvs.2014.06.023 [Accessed 23 January 2017]. | [Korea](http://dx.doi.org/10.1016/j.ejvs.2015.06.013) |
