# Supplementary material for: The comparative efficacy of angiosome-directed and indirect revascularisation strategies to aid healing of chronic foot wounds in patients with co-morbid diabetes mellitus and critical limb ischaemia: a literature review
Source: J Foot Ankle Res. 2017 Jun 28;10:26. doi: 10.1186/s13047-017-0206-5 (PMC5490238; doi:10.1186/s13047-017-0206-5)
Supplement: Supplementary file 3 — Full-text Articles Excluded. (DOCX 21 kb) [file 13047_2017_206_MOESM3_ESM.docx]

## Additional file 3: Full-text Articles Excluded

| **No** | **Citation** | **Country** | **Reason for exclusion** |
| --- | --- | --- | --- |
| 1 | Aerden D, Denecker N, Gallala S, Debing E, Van den Brande P. Wound Morphology and Topography in the Diabetic Foot: Hurdles in Implementing Angiosome-Guided Revascularization. *International Journal of Vascular Medicine*. 2014;2014(2014):1-6. Available from: doi: 10.1155/2014/672897 [Accessed 16 January 2016] | - Belgium | - Nil wound healing outcomes - No outcome data collected |
| 2 | Alexandrescu VA, Hubermont G, Philips Y, Guillaumie B, Ngongang C, Vandenbossche P, et al. Selective primary angioplasty following an angiosome model of reperfusion in the treatment of Wagner 1-4 diabetic foot lesions: practice in a multidisciplinary diabetic limb service. *Journal of Endovascular Therapy.* 2008;15(5):580-593. Available from: doi: 10.1583/08-2460.1 [Accessed 04 January 2016] | - Belgium | - Nil comparison between DR and IR |
| 3 | Alexandrescu V, Vincent G, Azdad K, Hubermont G, Ledent G, Ngongang C, et al. A reliable approach to diabetic neuroischemic foot wounds: below-the-knee angiosome-oriented angioplasty. *Journal of Endovascular Therapy*. 2011;18(3):376-387. Available from: doi: 10.1583/10-3260.1 [Accessed 04 January 2016] | - Belgium | - Mixed cohorts, i.e. inclusive of subjects with acute limb ischaemia |
| 4 | Azuma N, Uchida H, Kokubo T, Koya A, Akasaka N, Sasajima T. Factors Influencing Wound Healing of Critical Ischaemic Foot after Bypass Surgery: Is the Angiosome Important in Selecting Bypass Target Artery?. *European Journal of Vascular and Endovascular Surgery*. 2012;43(3):322-328. Available from: doi: 10.1016/j.ejvs.2011.12.001 [Accessed 02 January 2016] | - Japan | - Mixed cohorts, i.e. not all subjects have diabetes |
| 5 | Kabra A, Suresh KR, Vivekanand V, Vishnu M, Sumanth R, Nekkanti M. Outcomes of angiosome and non-angiosome targeted revascularization in critical lower limb ischemia. *Journal of Vascular Surgery.* 2013;57(1):44-49. Available from: doi: 10.1016/j.jvs.2012.07.042 [Accessed 04 January 2016] | - India | - Mixed cohorts, i.e. not all subjects have diabetes |
| 6 | Kagaya Y, Ohura N, Suga H, Eto H, Takushima A, Harii K. 'Real angiosome' assessment from peripheral tissue perfusion using tissue oxygen saturation foot-mapping in patients with critical limb ischemia. *European Journal of Vascular and Endovascular Surgery*. 2014;47(4):433-441. Available from: doi: 10.1016/j.ejvs.2013.11.011 [Accessed 04 January 2016] | - Japan | - Nil wound healing outcomes |
| 7 | Kret MR, Cheng D, Azarbal AF, Mitchell EL, Liem TK, Moneta GL, et al. Utility of direct angiosome revascularization and runoff scores in predicting outcomes in patients undergoing revascularization for critical limb ischemia. *Journal of Vascular Surgery*. 2014;59(1):121-128. Available from: doi: 10.1016/j.jvs.2013.06.075 [Accessed 02 January 2016] | - USA | - Mixed cohorts, i.e. not all subjects have diabetes |
| 8 | Lida O, Nanto S, Uematsu M, Ikeoka K, Okamoto S, Dohi T, et al. Importance of the angiosome concept for endovascular therapy in patients with critical limb ischemia. *Catheterization and Cardiovascular Interventions.* 2010;75(6):830-836. Available from: doi: 10.1002/ccd.22319 [Accessed 04 January 2016] | - Japan | - Mixed cohorts, i.e. not all subjects have diabetes - Nil wound healing outcomes |
| 9 | Lida O, Soga Y, Hirano K, Kawasaki D, Suzuki K, Miyashita Y, et al. Long-term results of direct and indirect endovascular revascularization based on the angiosome concept in patients with critical limb ischemia presenting with isolated below-the-knee lesions. *Journal of Vascular Surgery*. 2012;55(2):363-370.e5. Available from: doi: 10.1016/j.jvs.2011.08.014 [Accessed 04 January 2016] | - Japan | - Mixed cohorts, i.e. not all subjects have diabetes - Nil wound healing outcomes |
| 10 | Lida O, Takahara M, Soga Y, Yamauchi Y, Hirano K, Tazaki J, et al. Worse limb prognosis for indirect versus direct endovascular revascularization only in patients with critical limb ischemia complicated with wound infection and diabetes mellitus. *European Journal of Vascular and Endovascular Surgery.* 2013;46(5):575-582. Available from: doi: 10.1016/j.ejvs.2013.08.002 [Accessed 02 January 2016] | - Japan | - Mixed cohorts, i.e. not all subjects have diabetes - Nil wound healing outcomes |
| 11 | Neville RF, Attinger CE, Bulan EJ, Ducic I, Thomassen M, Sidawy AN. Revascularization of a specific angiosome for limb salvage: does the target artery matter?. *Annals of Vascular Surgery.* 2009;23(3):367-373. Available from: doi: 10.1016/j.avsg.2008.08.022 [Accessed 04 January 2016] | - USA | - Mixed cohorts, i.e. not all subjects have diabetes |
| 12 | Pavé M, Benadiba L, Berger L, Gouicem D, Hendricks M, Plissonnier D. Below-The-Knee Angioplasty for Critical Limb Ischemia: Results of a Series of 157 Procedures and Impact of the Angiosome Concept. *Annals Vascular Surgery*. 2016;36:199-207. Available from: doi: 10.1016/j.avsg.2016.03.032 [Accessed 24 January 2017] | - France | - Mixed cohorts, i.e. not all subjects have diabetes |
| 13 | Rashid H, Slim H, Zayed H, Huang DY, Wilkins CJ, Evans DR. The impact of arterial pedal arch quality and angiosome revascularization on foot tissue loss healing and infrapopliteal bypass outcome. *Journal of Vascular Surgery*. 2013;57(5):1219-1226. Available from: doi: 10.1016/j.jvs.2012.10.129 [Accessed 02 January 2016] | - UK | - Mixed cohorts, i.e. not all subjects have diabetes |
| 14 | Shiraki T, Iida O, Takahara M, Soga Y, Yamauchi Y, Hirano K, et al. Predictors of Delayed Wound Healing after Endovascular Therapy of Isolated Infrapopliteal Lesions Underlying Critical Limb Ischemia in Patients with High Prevalence of Diabetes Mellitus and Hemodialysis. *European Journal of Vascular and Endovascular Surgery*. 2015;49(5):565–573. Available from: doi: 10.1016/j.ejvs.2015.01.017 [Accessed 02 January 2016] | - Japan | - Nil comparison between DR and IR |
| 15 | Soares Rde A, Brochado Neto FC, Matielo MF, Lehn CN, Nakamura ET, Godoy MR, et al. Concept of Angiosome Does Not Affect Limb Salvage in Infrapopliteal Angioplasty. *Annals of Vascular Surgery.* 2016;32:34-40. Available at: doi: 10.1016/j.avsg.2015.09.024 [Accessed 29 October 2016] | - Brazil | - Mixed cohorts, i.e. not all subjects have diabetes |
| 16 | Špillerová K, Biancari F, Leppäniemi A, Albäck A, Söderström M, Venermo M. Differential Impact of Bypass Surgery and Angioplasty on Angiosome-Targeted Infrapopliteal Revascularization. *European Journal of Vascular and Endovascular Surgery*. 2015;49(4):412-491. Available from: doi: 10.1016/j.ejvs.2014.12.023 [Accessed 04 January 2016] | - Finland | - Nil comparison between DR and IR |
| 17 | Špillerová K, Sörderström M, Albäck A, Venermo M. The Feasibility of Angiosome-Targeted Endovascular Treatment in Patients with Critical Limb Ischemia and Foot Ulcer. *Annals of Vascular Surgery.* 2016;30:270-276. Available from: doi: 10.1016/j.avsg.2015.07.020 [Accessed 16 January 2016] | - Finland | - Nil wound healing outcome measures |
| 18 | Varela C, Acín F, de Haro J, Bleda S, Esparza L, March JR. The role of foot collateral vessels on ulcer healing and limb salvage after successful endovascular and surgical distal procedures according to an angiosome model. *Vascular and Endovascular Surgery*. 2010;44(8):654-660. Available from: doi: 10.1177/1538574410376601 [Accessed 16 January 2016] | - Spain | - Mixed cohorts, i.e. not all subjects have diabetes |
| 19 | Zheng XT, Zeng RC, Huang JY, Pan LM, Su X, Wu ZH, et al. The Use of the Angiosome Concept for Treating Infrapopliteal Critical Limb Ischemia through Interventional Therapy and Determining the Clinical Significance of Collateral Vessels. *Annals of Vascular Surgery.* 2016;32:41-49. Available from: doi: 10.1016/j.avsg.2015.09.021 [Accessed 29 October 2016] | - China | - Mixed cohorts, i.e. not all subjects have diabetes |
