# Supplementary material for: The comparative efficacy of angiosome-directed and indirect revascularisation strategies to aid healing of chronic foot wounds in patients with co-morbid diabetes mellitus and critical limb ischaemia: a literature review
Source: J Foot Ankle Res. 2017 Jun 28;10:26. doi: 10.1186/s13047-017-0206-5 (PMC5490238; doi:10.1186/s13047-017-0206-5)
Supplement: Supplementary file 4 — Literature Review Tables. (DOCX 34 kb) [file 13047_2017_206_MOESM4_ESM.docx]

## Additional file 4: Literature Review Tables Adapted from: SCOTTISH INTERCOLLEGIATE GUIDELINE NETWORK (SIGN), 2008. *SIGN 50 A guideline developer’s handbook*. NHS Quality Improvement Scotland [online]. [viewed 20 January 2016]. Available at: <http://www.sign.ac.uk/pdf/sign50.pdf>.

| **Bibliographic citation** | **Study type** | **No. of patients**  **(no. of limbs)** | **Patient characteristics** | **Intervention** | **Comparison #1** | **Comparison #2** | **Length of follow up (months)** | **Outcome measures** | **NOS score** | **Conflict of interest** |
| --- | --- | --- | --- | --- | --- | --- | --- | --- | --- | --- |
| Acín F, Varela C, de Maturana IL, de Haro J, Bleda S, Rodriguez-Padilla J. Results of infrapopliteal endovascular procedures performed in diabetic patients with critical limb ischemia and tissue loss from the perspective of an angiosome-oriented revascularization strategy. *International Journal of Vascular Medicine.* 2014;2014(2014):1-13. Available from: doi: 10.1155/2014/270539 [Accessed 04 January 2016] | - Spain - Single centre - Retrospective, non-randomised cohort study - Study period: 1999-2009 | 92 patients  (101 limbs) | - 100% with  CLI and diabetes - PAD anatomical lesions: Femoro-popliteal and infrapopliteal lesions - Wound classification:  (1) nil for ulcer classification; (2) presence of infection graded using the CDC / NHSN surveillance definition | - Angioplasty: primary endoluminal - Stents used selectively - DR - In 46 limbs      - Grouping method:  Cohort was divided according to the no. of tibial vessels attempted for treatment, the no. of patent tibial vessels finally achieved to the foot and the local perfusion of the ischemic ulcer obtained after revascularisation. | - Angioplasty: primary endoluminal - Stents used selectively - IR ‘through collaterals’ - In 22 limbs      - Grouping method:  Cohort was divided according to the no. of tibial vessels attempted for treatment, the no. of patent tibial vessels finally achieved to the foot and the local perfusion of the ischemic ulcer obtained after revascularisation | - Angioplasty: primary endoluminal - Stents used selectively - IR ‘without collaterals’ - In 17 limbs      - Grouping method:  Cohort was divided according to the no. of tibial vessels attempted for treatment, the no. of patent tibial vessels finally achieved to the foot and the local perfusion of the ischemic ulcer obtained after revascularisation | 1,3, and every 6 months thereafter  Median: 19  Range: 9–38  No. of patients lost to follow-up: 11 (10.8%) | - Wound healing - Limb salvage - Overall survival at 24 months - Major amputation at 30 days - Amputation-free survival - Major adverse cardiovascular event (MACE) - Major adverse limb event (MALE) - Freedom from MALE+ POD (perioperative death) - Freedom from RAS (restenosis, any reintervention or amputation) - Freedom from RAO (reintervention or amputation) | 5/9 | None  declared |
| ***General comments:***   - **Strengths**: (1) TASC-II diagnostic criteria (Norgren et al., 2007) for CLI satisfied; (2) Diagnostic criteria of diabetes indicated; (3) Consecutive sample; (4) Presence of infection accounted for; (5) Comparable baseline characteristics of subjects between DR and IR groups. - **Limitations**: (1) No data on subject’s duration of diabetes; (2) Drop-outs unaccounted for; (3) Wound classification system not utilised; (4) Patients with end-stage renal disease excluded. | | | | | | | | | | |

| **Bibliographic citation** | **Study type** | **Number of patients**  **(no. of limbs)** | **Patient characteristics** | **Intervention** | **Comparison** | **Length of follow up** | **Outcome measures** | **NOS score** | **Conflict of interest** |
| --- | --- | --- | --- | --- | --- | --- | --- | --- | --- |
| Fossaceca R, Guzzardi G, Cerini P, Cusaro C, Stecco A, Parziale G, et al. Endovascular treatment of diabetic foot in a selected population of patients with below-the-knee disease: is the angiosome model effective?. *CardioVascular and Interventional Radiology.* 2013;36(3):637-644. Available from: doi: 10.1007/s00270-012-0544-4 [Accessed 04 January 2016] | - Italy - Single centre - Retrospective, non-randomised cohort study - Study period:  2005-2011 | 201 patients  (201 limbs) | - 100% with  CLI and diabetes - PAD anatomical distribution: Isolated below-the-knee lesions - Wound classification:  (1) nil for ulcer classification; (2) nil for infection status | - Angioplasty: primary endoluminal, secondary subintimal approach - DR - In 167 limbs - Grouping method:  Angiosome concept.  All patients primarily considered for DR. | - Angioplasty: primary endoluminal, secondary subintimal approach - IR - In 34 limbs - Grouping method: Angiosome concept. Patients subsequently underwent IR when all DR options were not technically feasible. | 1, 6, 12 months | - Wound healing (partial and complete) - Limb salvage - Amputation (minor and major) - Average TcPO_2_ - Mortality - PTA retreatment - Restenosis - Technical success | 6/9 | None  declared |
| **General comments:**   - **Strengths:** (1) TASC-II diagnostic criteria (Norgren et al., 2007) for CLI satisfied; (2) Complete follow-up of all subjects; (3) Diagnostic criteria for diabetes indicated; (4) Subjects’ duration of diabetes provided, along with HbA1c levels and number of patients on insulin therapy. - **Limitations:** (1) Non-consecutive sample; (2) Wound classification system not utilised; (3) Presence of infection not accounted for; (4) Omission of subjects’ baseline characteristics. | | | | | | | | | |

| **Bibliographic citation** | **Study type** | **Number of patients**  **(no. of limbs)** | **Patient characteristics** | **Intervention** | **Comparison** | **Length of follow up** | **Outcome measures** | **NOS score** | **Conflict of interest** |
| --- | --- | --- | --- | --- | --- | --- | --- | --- | --- |
| Lejay A, Georg Y, Tartaglia E, Gaertner S, Geny B, Thaveau F, et al. Long-term outcomes of direct and indirect below-the-knee open revascularization based on the angiosome concept in diabetic patients with critical limb ischemia. *Annals of Vascular Surgery.* 2014;28(4):983-989. Available from: doi: 10.1016/j.avsg.2013.08.026 [Accessed 02 January 2016] | - France - Single centre - Retrospective, non-randomised cohort study - Study period: 2003-2009 | 54 patients  (58 limbs) | - 100% with  CLI and diabetes - PAD anatomical distribution: isolated below-the-knee lesions - Wound classification: (1) Diabetic foot Armstrong classification (i.e. UTWCS); (2) Presence of infection accounted for and appropriately categorised in further subgroups. | - Bypass: autologous saphenous vein conduits only - DR - In 36 limbs (62%)      - Grouping method:  Angiosome concept.  All patients primarily considered for DR. | - Bypass: autologous saphenous vein conduits only - IR - In 22 limbs (38%)      - Grouping method: Angiosome concept. Patients subsequently underwent IR when all DR options were not technically feasible. | 1, 3, 6, every 6 months thereafter  Mean: 20 ± 16 months | - Wound healing - Limb salvage - Median ulcer-healing time - Primary patency - Survival - TcPO_2_ | 7/9 | No data. |
| **General comments:**   - **Strengths:** (1) TASC-II diagnostic criteria (Norgren et al., 2007) for CLI satisfied; (2) Complete follow-up of all subjects; (3) Consecutive sample; (4) Employment of wound classification system; (5) Presence of infection accounted for; (6) Comparable baseline characteristics of subjects between DR and IR groups. - **Limitations:** (1) No data on diagnostic criteria for diabetes; (2) No data on subjects’ duration of diabetes. - **Additional details of study**: (1) If the foot ulcer recurred within 3 months of complete epithelialisation, the diagnosis of wound healing was rescinded; (2) Wound care could not be strictly standardised due to ethical reasons. | | | | | | | | | |

| **Bibliographic citation** | **Study type** | **Number of patients**  **(no. of limbs)** | **Patient characteristics** | **Intervention** | **Comparison** | **Length of follow up** | **Outcome measures** | **NOS score** | **Conflict of interest** |
| --- | --- | --- | --- | --- | --- | --- | --- | --- | --- |
| Jeon EY, Cho YK, Yoon DY, Kim DJ, Woo JJ. Clinical outcome of angiosome-oriented infrapopliteal percutaneous transluminal angioplasty for isolated infrapopliteal lesions in patients with critical limb ischemia. *Diagnostic and Interventional Radiology.* 2016;22(1):52-58. Available from: doi: 10.5152/dir.2015.15129 [Accessed 27 August 2016] | - South Korea - No. of centres unspecified - Retrospective, non-randomised cohort study - Study period: 2011-2013 | 70 patients  (82 limbs) | - 100% with  CLI and diabetes - PAD anatomical distribution: isolated infrapopliteal lesions - Wound classification:  (1) Wagner ulcer classification; (2) nil for infection status | - Angioplasty: primary intraluminal, secondary subintimal approach - DR - In 63 limbs - Grouping method:  Angiosome concept.  All patients primarily considered for DR. | - Angioplasty: primary intraluminal, secondary subintimal approach - DR - In 19 limbs - Grouping method:  Angiosome concept. Patients subsequently underwent IR when all DR options were not technically feasible. | 12, 24 months  Mean: 13 (range: 0-25) | - Wound healing - Limb salvage - Amputation - Angiosome score - Major and minor complications - Mortality - PTA re-intervention - Technical success - Wound healing time | 5/9 | None  declared |
| **General comments:**   - **Strengths:** (1) TASC-II diagnostic criteria (Norgren et al., 2007) for CLI satisfied; (2) Diagnostic criteria for diabetes indicated; (3) Subjects’ duration of diabetes provided; (4) Employment of wound classification system - **Limitations:** (1) Drop-outs unaccounted; (2) Non-consecutive sample; (3) Presence of infection not accounted for; (4) Omission of subjects’ baseline characteristics. | | | | | | | | | |

| **Bibliographic citation** | **Study type** | **Number of patients**  **(no. of limbs)** | **Patient characteristics** | **Intervention** | **Comparison** | **Length of follow up** | **Outcome measures** | **NOS score** | **Conflict of interest** |
| --- | --- | --- | --- | --- | --- | --- | --- | --- | --- |
| Söderström M, Albäck A, Biancari F, Lappalainen K, Lepäntalo M, Venermo M. Angiosome-targeted infrapopliteal endovascular revascularization for treatment of diabetic foot ulcers. *Journal of Vascular Surgery.* 2013;57(2):427-435. Available from: doi: 10.1016/j.jvs.2012.07.057 [Accessed 02 January 2016] | - Finland - Single centre - Retrospective, non-randomised cohort study - Study period: 2007-2011 | 226 patients  (250 limbs) | - 100% with  CLI and diabetes - PAD anatomical lesions: isolated infrapopliteal lesions - Wound classification: (1) University of Texas Wound Classification System (UTWCS); (2) Presence of infection accounted for, appropriately categorised in further subgroups. | - Angioplasty:  primary intraluminal - DR - In 121 limbs (48%) - Grouping method: Best vessel strategy. Retrospective grouping of patients into DR if the best vessel utilised supplied the ischaemic site via a source artery. - Where the foot ulcer spans numerous angiosome territories, the limb was grouped under DR if direct flow to the ulcer was successfully achieved from at least one crural artery. | - Angioplasty: primary intraluminal - IR - In 129 limbs (52%) - Grouping method: Best vessel strategy. Retrospective grouping of patients into IR if the best vessel utilised supplied the ischaemic site via collaterals.  Where the foot ulcer spans numerous angiosome territories, the limb was grouped under IR if direct flow to the ulcer could not be achieved from at least one crural artery. | 1 month, and at 1-3 months thereafter depending on clinical status of the foot.  Follow-up ended 1 year after the intervention or death, whichever occurred first. | - Ulcer healing - Limb salvage - Amputation-free survival - Amputation-free survival with healed ulcer - Median time to ulcer healing - Survival - Vascular re-intervention | 8/9 | None  declared de declared de |
| **General comments:**   - **Strengths:** (1) TASC-II diagnostic criteria (Norgren et al., 2007) for CLI satisfied; (2) Complete follow-up of all subjects; (3) Diagnostic criteria of diabetes indicated; (4) Consecutive sample; (5) Employment of wound classification system; (6) Presence of infection accounted for; (7) Propensity score utilised. - **Limitations:** (1) No data on subjects’ duration of disease. - **Additional details of study**: (1) Wound care could not be strictly standardised due to ethical reasons. | | | | | | | | | |
