# Supplementary material for: The comparative efficacy of angiosome-directed and indirect revascularisation strategies to aid healing of chronic foot wounds in patients with co-morbid diabetes mellitus and critical limb ischaemia: a literature review
Source: J Foot Ankle Res. 2017 Jun 28;10:26. doi: 10.1186/s13047-017-0206-5 (PMC5490238; doi:10.1186/s13047-017-0206-5)
Supplement: Supplementary file 5 — The Newcastle-Ottawa Scale (NOS) scores. (DOCX 20 kb) [file 13047_2017_206_MOESM5_ESM.docx]

## Additional file 5: The Newcastle-Ottawa Scale (NOS) scores [adapted from 31]

| Newcastle-Ottawa Scale: Quality assessment criteria | Acceptable (★) | Not acceptable | Fossaceca et al., 2013 [36] | Söderström et al., 2013 [37] | Acín et al., 2014 [38] | Lejay et al., 2014 [39] | Jeon et al., 2016 [40] |
| --- | --- | --- | --- | --- | --- | --- | --- |
| **Selection** | | | | | | | |
| 1. Representative-ness of the exposed cohort | Truly or somewhat representative of the average diabetic patient with CLI | - Selected group of users (e.g. volunteers) - No description of the derivation of the cohort | -  No description of baseline characteristics of cohort | ★ somewhat  Patients who previously underwent infrainguinal revascularisation excluded. | -  Patients with end-stage renal disease excluded | ★somewhat Only patients who underwent saphenous vein conduits included | -  No description of baseline characteristics of cohort |
| 1. Selection of the comparison cohort | Drawn from the same community as the intervention cohort | - Drawn from a different source - No description of the derivation of the comparison cohort | ★ | ★ | ★ | ★ | ★ |
| 1. **Ascertainment of exposure** | - Secure record (e.g. surgical records) - Structured interview | - Written self report - No description | ★ | ★ | ★ | ★ | ★ |
| 1. Demonstration that outcome of interest was not present at start of study | Yes | No | ★ | ★ | ★ | ★ | ★ |
| 1. Comparability of cohorts on the basis of the design or analysis   (a max of 2 starts can be allotted in this category)   - *Either exposed and non-exposed individuals must be matched in the design and/or confounders must be adjusted for in the analysis.* - *Statements of no differences between groups or that differences were not statistically significant are not sufficient for establishing comparability.* | - a) study controls for differences in baseline characteristics of groups - b) study controls for any other additional factors | Study did not control for any factors. | - | ★ – Overt baseline disparities adjusted using propensity score | - | - | - |
| **Outcome** | | | | | | | |
| 1. Assessment of outcome | - Independent blind assessment - Record linkage | - Self report - No description | ★ - record linkage | ★ - record linkage | ★ - record linkage | ★ - record linkage | ★ - record linkage |
| 1. Was follow-up long enough for outcomes to occur? | Follow-up ≥1 year | Follow-up <1 year | ★ - 1 year | ★ - 1 year | ★ - 1 year | ★ - 1 year | ★ - 1 year |
| 1. Adequacy of follow up of cohorts | - Complete follow up - all subjects accounted for - Small number of subjects lost to follow up unlikely to introduce bias | - High rate of subjects lost to follow up likely to introduce bias - No statement | ★- Complete follow-up | ★- Complete follow-up | **-**  11 (10.8%) patients lost to follow-up | ★- Complete follow-up | **-**  21 (15.5%) patients lost to follow-up |
| 1. Overall quality score | **(Maximum 9)** | | 6 | 8 | 5 | 7 | 5 |
